# Protocol for a confirmatory trial of the effectiveness and safety of palliative arterial embolization for painful bone metastases

**DOI:** 10.1186/s12885-023-10538-6

**Published:** 2023-01-31

**Authors:** Sadamoto Zenda, Yasunori Arai, Shunsuke Sugawara, Yoshitaka Inaba, Kazuki Hashimoto, Kouji Yamamoto, Yusuke Saigusa, Takashi Kawaguchi, Sanae Shimada, Marie Yokoyama, Tempei Miyaji, Tomoka Okano, Naoki Nakamura, Eisuke Kobayashi, Tatsuya Takagi, Yoshihisa Matsumoto, Yosuke Uchitomi, Miyuki Sone, for the J-SUPPORT 1903, PALEM Trial

**Affiliations:** 1 Department of Radiation Oncology, National Cancer Center Hospital East, 6-5-1 Kashiwanoha, Kashiwa Chiba, Japan; 2 Department of Supportive and Palliative Care Research Support Office, National Cancer Center Hospital East, 6-5-1 Kashiwanoha, Kashiwa Chiba, Japan; 3 Department of Radiology, National Cancer Center Hospital East, 6-5-1 Kashiwanoha, Kashiwa Chiba, Japan; 4 Department of Radiology, National Cancer Center Hospital East, 5-1-1 Tsukiji Chuo-Ku, Tokyo, Japan; 5 Department of Diagnostic and Interventional Radiology, Aichi Cancer Center, 1-1, Kanokoden, Chikusa-Ku, Nagoya, Aichi 464-8681 Japan; 6 Department of Radiology, St. Marianna University School of Medicine, Sugao 2-16-1, Miyamae, Kawasaki, Kanagawa Japan; 7 Department of Biostatistics, Yokohama City University, 3-9 Fukuura Kanazawa-Ku, Yokohama City, Kanagawa Japan; 8 Department of Practical Pharmacy, Tokyo University of Pharmacy and Life Sciences, 1432-1 Horinouchi Hachioji, Tokyo, Japan; 9 Division of Supportive Care, Survivorship and Translational Research, Institute for Cancer Control, National Cancer Center, 5-1-1 Tsukiji, Tokyo, 104-0045 Japan; 10 Department of Radiation Oncology, St. Marianna University School of Medicine, Sugao 2-16-1, Miyamae, Kawasaki, Kanagawa Japan; 11 Department of Musculoskeletal Oncology, National Cancer Center Hospital, 5-1-1 Tsukiji Chuo-Ku, Tokyo, Japan; 12 Department of Orthopedic Surgery, Juntendo University School of Medicine, 3-1-3 Hongo Bunkyo-Ku, Tokyo, Japan; 13 Department of Palliative Care, Cancer Institute Hospital of JFCR, 3-8-31 Ariake Koto-Ku, Tokyo, Japan

**Keywords:** Painful bone metastases, Arterial embolization, Palliative care, Confirmatory study

## Abstract

**Background:**

Transcatheter arterial embolization (TAE) has long been used for hemostasis of traumatic or postoperative hemorrhage and embolization of tumors. Previous retrospective studies of TAE for painful bone metastases showed 60%–80% pain reduction with a median time to response of 1–2 days. Compared with radiotherapy and bisphosphonates, time to response appeared earlier than that of radiotherapy or bone-modifying agents. However, few prospective studies have examined TAE for this indication. Here, we describe the protocol for a confirmatory study designed to clarify the efficacy and safety profile of TAE.

**Methods:**

This study will be a multicenter, single-arm confirmatory study (phase 2–3 design). Patients with painful bone metastases from any primary tumor are eligible for enrollment. TAE will be the main intervention. Following puncture of the femoral artery under local anesthesia and insertion of an angiographic sheath, angiography will confirm that the injected region includes tumor vasculature. Catheter position will be adjusted so that the embolization range does not include non-target tissues. Spherical embolic material will then be slowly injected into the artery to embolize it. The primary endpoint (efficacy) is the proportion of subjects with pain relief at 72 h after TAE and the secondary endpoint (safety) is the incidence of all NCI Common Terminology Criteria for Adverse Events version 5.0 Grade 4 adverse events and Grade ≥ 3 necrosis of the central nervous system.

**Discussion:**

If the primary and secondary endpoints are met, TAE can be a treatment choice for painful bone metastases. Trial registry number is UMIN-CTR ID: UMIN000040794.

**Trial registration:**

The study is ongoing, and patients are currently being enrolled. Enrollment started in March 2021. A total of 36 patients have participated as of Aug 2022.

Protocol Version: Ver1.4, 13/07/2022.

## Background

As life expectancy increases due to recent advances in cancer treatment, and the number of cancer patients increases due to an increase in cancer incidence resulting from an increase in patients surviving stroke and other cardiovascular disease, the number of patients with bone metastases is also increasing [1]. It is reported that approximately 75% of bone metastases are painful [2].

For patients with painful bone metastases, there are different approaches to pain relief. Among current standard treatments, time to pain relief is 4 to 8 weeks in most patients receiving radiotherapy [3, 4] and 1 to 3 months in responders to bone-modifying agents [5] (30% of treated patients). Surgery improves symptoms quite rapidly, but is indicated for limited pathologies and is often unfeasible due to its invasiveness, depending on the patient’s general condition [6]. Analgesics, including opioids, temporarily relieve pain but do not remove its cause.

Arterial embolization is used for hemostasis of traumatic or postoperative hemorrhage and for embolization to reduce tumor size by ischemia. Previous retrospective studies of arterial embolization for painful bone metastases [7–11] showed pain reduction of 60%–80% with a median time to response of 1–2 days. Compared with radiotherapy and bone-modifying agents, this reported time to response is faster. Although arterial embolization with a mixture of thrombotic and chemotherapeutic agents, such as trans-arterial chemoembolization (TACE) for hepatocellular carcinoma, is a commonly used technique, few prospective studies have evaluated transcatheter arterial embolization (TAE) without chemotherapeutic agents for painful bone metastases.

Here, we report the protocol for a confirmatory study of TAE, which aims to clarify the efficacy and safety profiles of this treatment.

## Methods

### Study design

The purpose of this study is to clarify the efficacy and safety profile of TAE for painful bone metastases. This study is a collaboration between the Japan Interventional Radiology in Oncology Study Group (JIVROSG) and the Japan Supportive, Palliative, and Psychosocial Oncology Group (J-SUPPORT). This protocol has been reviewed by the J-SUPPORT Scientific Advisory Board, and approved as a JIVROS/J-SUPPORT1903 study (PALEM study).

### Patient and public involvement

Cancer survivors and nonmedical persons were invited to attend the scientific advisory board meeting of J-SUPPORT. From protocol concept to writing the informed consent document, they participated in our discussion. Scientific advisory board members are listed on the following web page: Committees and Administrative Office | J-SUPPORT.

### Study setting

This study is a multicenter, single-arm confirmatory study to clarify the efficacy and safety profile of TAE for painful bone metastases (phase 2–3 design).

All participants will be recruited from 24 hospitals: Aichi Medical College, Aichi Cancer Center, Iwate Medical College, Cancer Institute Hospital of JFCR, Tonan Hospital, National Cancer Center Hospital, National Cancer Center Hospital East, Shizuoka Cancer Center, Juntendo Hospital, Teina Keijinkai Hospital, Hokkaido University Hospital, Ryukyu University Hospital, Osaka University, Kobe University, Keio University, Saiseikai Fukuoka Sogo Hospital, Nara City Hospital, Hakodate Goryokaku Hospital, Hyogo Cancer Center, Hyogo Medical University, Fukuchiyama City Hospital, Nara Medical Hospital, St. Marianna University Hospital, and Hokkaido Cancer Center.

The protocol treatment will be performed on inpatients.

**Fig. 1:**
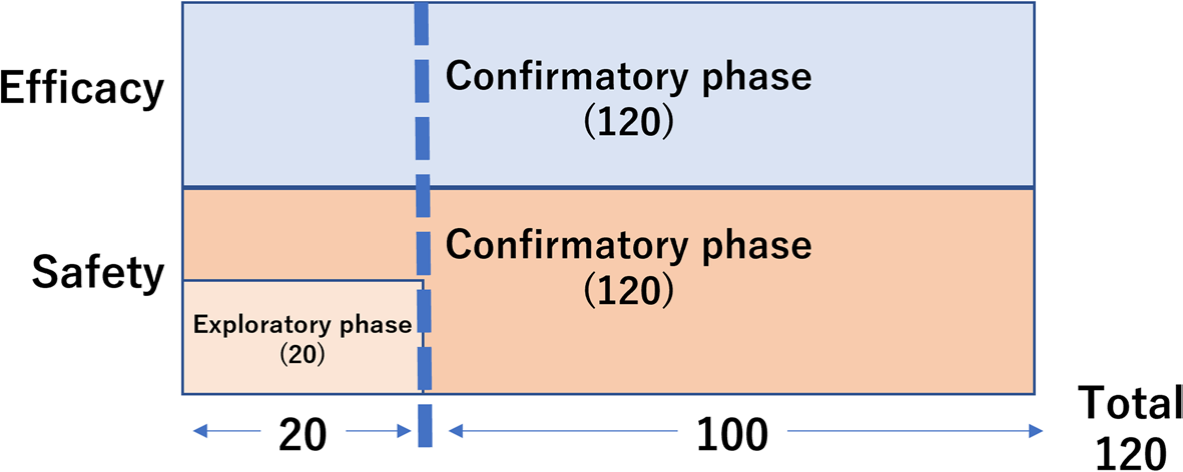
Study schema this study consists of two sections, the exploratory and confirmatory phases. Registration will be suspended when 20 subjects are recruited to analyze the data set rather than investigate all the events individually. (Stopping rule) Recruitment of a total of 120 patients is planned

To ensure the safety of subjects, registration will be suspended to evaluate safety once 20 subjects are recruited. In the exploratory phase, if any CTCAE Grade 4 adverse event or a Grade 3 or higher adverse event of central nervous system (CNS) necrosis occurs in at least 4 of the 20 subjects, the study will be discontinued (stopping rule) (Fig. 1).

#### Inclusion criteria

Patients fulfilling all the following criteria will be eligible for inclusion in this study:

- 1) Diagnosis of painful bone metastases (multiple metastases are acceptable)
- 2) The most painful bone metastases involve the thoracic spine, lumbar spine, pelvis, or rib(s), for which arterial embolization is expected to relieve pain
- 3) No evidence of spinal cord compression on CT or MRI scan of the most painful bone metastases
- 4) Numerical rating scale (NRS) score of ≥ 5 (Max10) for bone metastatic pain
- 5) Current use of opioid analgesics
- 6) No radiotherapy (RT) at the planned site(s) of TAE within the preceding 3 months
- 7) No initiation of new chemotherapy or hormone therapy within the preceding 2 weeks
- 8) Patients with any cancer
- 9) Life expectancy > 1 month
- 10) Normal renal function and no bleeding risk
- 11) Age ≥ 20 years
- 12) Ability to provide written informed consent to participate in the study

#### Exclusion criteria

Patients fulfilling any of the following criteria will be excluded from this study:

- 1) Inability to remain in the supine position
- 2) Inability to describe pain on an NRS [12, 13]
- 3) History of contrast media allergy
- 4) Inability to discontinue anticoagulants, antithrombotics, or antiplatelet medication other than aspirin
- 5) Childbearing potential
- 6) Inappropriate for enrollment in the opinion of the investigator or sub-investigator

### Intervention for bone metastases

The study treatment will be performed in an angiography suite on an inpatient basis. An angiography device that can be used in digital subtraction angiography (DSA) is necessary. It is desirable that cone beam CT and/or CT angiography are available.

**Fig. 2:**
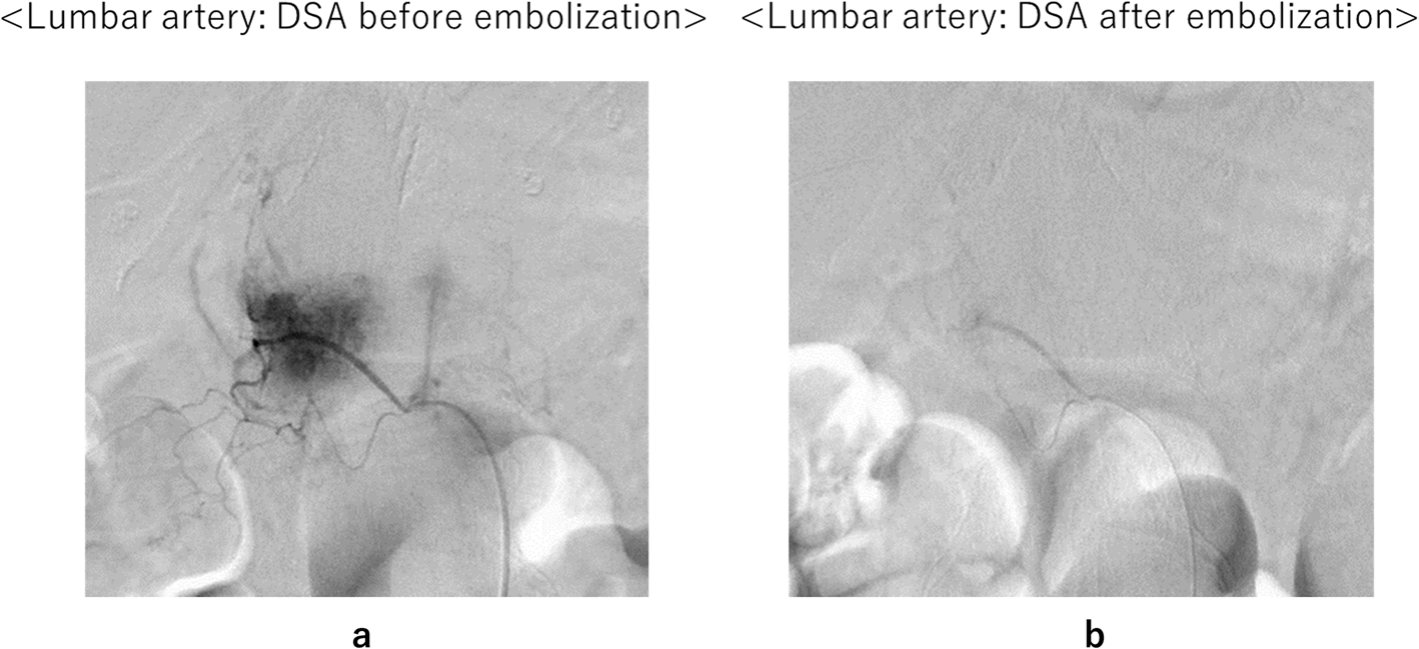
Example of arterial embolization **a**. Lumbar artery: DSA before embolization **b**. Lumbar artery: DSA after embolization

As with conventional angiography, the femoral artery is punctured under local anesthesia to insert an angiographic sheath. Using an imaging catheter (parent catheter: 3 to 5 Fr) from the sheath, a parent vessel of the feeding artery is selected and imaged to localize the tumor and determine the vascular morphology. A microcatheter (1.7 to 2.5 Fr) is then inserted through the parent catheter to select arteries feeding the target lesion. After selecting the feeding arterial branches, angiography is performed to confirm that the relevant region includes abundant tumor stain, but not blood vessels in which embolization is contraindicated. The catheter position is adjusted so that the embolization range does not affect non-target tissues. Spherical embolic material is then slowly injected into the artery to embolize it (Fig. 2). In Japan, spherical embolic material was approved for insurance coverage in 2013, and four products (Embosphere®, BioSphere Medical, Rockland MA, USA), DC Bead® (Biocompatibles UK, Surrey, UK), HepaSphere® (BioSphere), and Embozene® (CeloNova Biosciences, Newnan GA, USA) are commercially available as of 2020.

In this study, spherical embolic material will be primarily used to embolize arteries feeding the tumor. Spherical embolic material has been clinically applied since it was commercialized in Europe in the early 1990s. This material is characterized by its reliable embolization of target blood vessels, because it is spherical with uniform diameter and does not aggregate. In this regard, it is unlike conventional gelatin preparations and polyvinyl alcohol (PVA), which are irregular in shape and diameter. It allows embolization of blood vessels of a specific target size with less proximal embolization, and contains fewer proinflammatory raw materials.

### Permitted concomitant therapies

All cancer treatments (including chemotherapy, immunotherapy, and Chinese medicine) that have been used since before enrollment in the study can be continued. Hydroxyzine, pentazocine, opioid analgesics, dexmedetomidine, and midazolam can be used as premedication before TAE. For post-embolization syndrome following arterial embolization, NSAIDs, acetaminophen, rescue opioid analgesics, and adjuvant analgesics can be used for transient exacerbation of pain, and antiemetics can be used for nausea.

### Prohibited concomitant therapies

Subjects may not start any new cancer treatment (including surgery, chemotherapy, molecularly-targeted drugs, therapeutic antibodies, hormone therapy, immunotherapy, Chinese medicine, or radiotherapy) between enrollment in the study and assessment at 2 weeks after arterial embolization.

### Endpoints

#### Efficacy

The primary endpoint (efficacy) is the proportion of subjects with pain relief at 72 h after TAE.

Compared with standard treatments for painful bone metastases, TAE is expected to produce a much more rapid response. Good pain relief may be achieved very rapidly after the start of palliative care by combining current standard treatments with TAE. Therefore, the proportion of subjects with pain relief as early as 72 h post-treatment is selected as the primary endpoint for immediate efficacy.

The assessment method and response assessment will be conducted in accordance with the International Consensus on Palliative Radiotherapy Endpoints, an international consensus proposed for clinical trials of palliative radiotherapy for bone metastases [14]. A responder is defined as a subject with a complete response (CR) or partial response (PR).

### Safety (secondary endpoint)

The secondary endpoint (safety) is the incidence of all CTCAE Grade 4 adverse events and CTCAE Grade ≥ 3 CNS necrosis associated with the arterial embolization procedure.

In this study, the incidence of all CTCAE Grade 4 adverse events and CTCAE Grade ≥ 3 CNS necrosis associated with the arterial embolization procedure will be assessed in the 2-week post-TAE observation period.

### Secondary endpoints

#### Incidence of adverse events associated with arterial embolization

The incidence of all CTCAE Grade ≥ 2 adverse events associated with TAE will be investigated. Further, arterial embolization for painful bone metastases is not technically difficult and is thus feasible for clinical introduction; given that this may be a key point, the proportion of subjects treated with arterial embolization is included as another secondary endpoint.

The incidence of adverse events will be calculated using the following equation:

1 $$incidence of adverse events = number of subjects with at least one adverse event/ Safety analysis set (SAS)$$

#### Proportion of subjects treated with TAE

The proportion of subjects treated with arterial embolization will be calculated using the following equation:

2 $$proportion of subjects treated with arterial embolization = (SAS - subjects discontinued from treatment)/SAS$$

#### Proportion of subjects with pain relief at 24 h/7 days/14 days after arterial embolization

To investigate the timing, course, and duration of pain relief in response to arterial embolization, the proportions of subjects with pain relief at 24 h, 7 days, and 14 days are included as other secondary endpoints. The proportion of subjects with pain relief will be assessed based on the Brief-Pain Inventory-Short Form (BPI-SF) questionnaire [15].

The proportion of subjects with pain relief will be calculated using the following equation:

3 $$proportion of subjects with pain relief = (complete response + partial response)/(FAS - subjects with missing data)$$

where “FAS” stands for “Full Analysis Set,”

#### Proportion of subjects with an improvement in Karnofsky Performance Score (KPS)

An improvement in KPS would provide an additional reason to use TAE instead of other methods. The proportion of subjects with an improvement in KPS is included as another secondary endpoint. An increase or decrease of ≥ 1 level will be counted as a significant change.

#### Proportion of subjects with a complete response

To demonstrate that TAE is more effective than placebo, the proportion of complete responders will be assessed.

The proportion of complete responders will be calculated using the following equation:

4 $$proportion of complete responders = complete response/(FAS - subjects with missing data)$$

The definition of an analysis set is the population of subjects enrolled in the study, excluding those who are doubly or wrongly registered (Table 1).

**Table 1:** The definition of each analysis set

Analysis set	Definition
All enrolled subjects	Population of subjects enrolled in the study, excluding those who are doubly or wrongly registered
Full analysis set (FAS)	Population of all enrolled subjects, excluding the following subjects:
	● Subjects who are found ex post facto to be ineligible (fail to meet all of the eligibility criteria or meet any of the exclusion criteria) based on information obtained in accordance with the protocol
	● Subjects who are found to be ineligible based on information obtained after enrollment or unscheduled examination results
	● Subjects who have never received transcatheter arterial embolization (non-implementation)
	● Subjects from whom no data have been collected after enrollment, except information at enrollment into the study
Safety analysis set (SAS)	Population of all enrolled subjects who have been treated with arterial embolization at least once
Per protocol set (PPS)	Population of subjects in the FAS who are in compliance with the protocol

#### Proportion of subjects with motion pain relief

In addition, since pain with motion is generally uncontrollable, it would be clinically significant to demonstrate an improvement in this variable with TAE. The proportion of subjects with decreased pain with motion will be assessed based on the BPI-SF questionnaire. It will be comprehensively determined based on the improvement in maximum pain measured by Item 3 and the improvement in motion measured by Item 9.

### Participant timeline

Patients will be registered online before the TAE protocol is initiated.

After protocol treatment starts, several evaluations for pain and a general examination will be performed at 24 h, 72 h, 7 days, and 14 days after protocol treatment. The assessment schedule from enrollment to 2 weeks after protocol treatment is shown in Table 2.

**Table 2:** Time Schedule

	Pretreatment	Arterial embolization	24 h (± 3 h)	72 h (± 3 h)	7 d (± 1 d)	14 d (± 2 d)
Karnofsky Performance Status (KPS)	〇		〇	〇	〇	〇
Numerical rating scale (NRS)	〇					
Brief Pain Inventory			〇	〇	〇	〇
Quantity of morphine used per day	〇		〇	〇	〇	〇
Blood exam	〇		〇			
CTCAE ver 5.0 (safety profile)		〇	→ always checked →			〇

### Sample size

The clinical hypothesis is that patients with painful bone metastases can be safely treated with arterial embolization for immediate pain relief.

The early response rate after arterial embolization must exceed that after radiotherapy in order for arterial embolization to be established as a new palliative therapy. Since the early response rate was reported to be 26% at 1 week after radiotherapy [16], it is assumed to be lower at 72 h after radiotherapy.

Assuming that the threshold for the proportion of subjects with pain relief at 72 h after arterial embolization, i.e., the primary efficacy endpoint, is 26%, and the expected response rate is 40%, which represents the minimum clinical utility, at least 113 patients are required to provide a power of 90% at a one-sided significance level of 2.5% (normal approximation test).

On the other hand, an adverse event associated with TAE, which is the primary safety endpoint, is defined as any CTCAE Grade 4 adverse event or Grade ≥ 3 CNS necrosis. As described in “Common Adverse Events Associated with Arterial Embolization,” adverse events (including mild events) have been reported in 5% to 7% of patients who undergo arteriography, 10% of patients treated with arterial embolization, and approximately 15% of patients treated with TACE. Considering that patients in poor general condition will be included in this study, the unacceptable threshold incidence for the clinical use of this treatment is set at 15%, equal to that for TACE for hepatocellular carcinoma [17]. Assuming that the incidence will actually be approximately 5% in this study where no anticancer drugs will be used concomitantly; and that bones will be treated, at least 96 patients are required to provide a power of 90% at a one-sided significance level of 2.5% (normal approximation test) (Fig. 1).

### Allocation

Not applicable.

### Masking

This is not a blinded study for either clinicians or patients.

### Data collection methods

The investigators will maintain individual records for each patient as source data, including a copy of informed consent, medical records, laboratory data, image data, patient diary, and other records or notes. All data will be collected by the J-SUPPORT Data Center at the National Cancer Center. Clinical data entry, data management, and central monitoring will be performed using an electronic data capture (EDC) system, Viedoc® 4 (PCG Solutions, Uppsala, Uppsala Lan, Sweden). Patient-reported outcomes will be collected using ePRO, Viedoc® Me (PCG Solutions).

### Statistical methods of analysis

As defined in the Section “Primary Endpoints,” the proportion of subjects with pain relief 72 h after arterial embolization and its two-sided 95% confidence interval (Clopper-Pearson method) will be calculated. It will be determined whether the lower limit of the two-sided 95% confidence interval is > 26%, the threshold response rate established at the time of study design. For reference, the *p*-value will be calculated by a binomial test.

Safety will be evaluated in the Safety Analysis Set (SAS) (Table 2). As defined in “Safety,” the incidence of all CTCAE Grade 4 adverse events and CTCAE Grade ≥ 3 adverse events of CNS necrosis associated with TAE and its two-sided 95% confidence interval (CI) (Clopper-Pearson method) will be calculated. It will be determined whether the upper limit of the two-sided 95% CI is < 15%, the threshold incidence established at the time of study design. For reference, the *p*-value will be calculated by a binomial test.

### Handling of missing values and outliers

In principle, all analyses will be performed without imputation of missing values or outliers. However, if it is found before data lock that missing values or outliers may significantly affect analysis results, countermeasures will be described in a statistical analysis plan.

## Discussion

In this study, we will examine the effect of TAE in providing immediate pain relief (within 3 days) for painful bone metastases. Palliative radiotherapy is one of the most commonly used modalities for painful bone metastases; however, its median time to pain relief is 4–8 weeks. Painkillers, including opioid analgesics, temporarily relieve pain but do not remove its cause. Therefore, a modality which can achieve immediate pain relief and remove its cause is necessary in this field. TAE may have the potential to achieve these goals.

Since there is no standard treatment to compare with TAE for relieving bone metastatic pain in as short as a few days, this study will be conducted with a single arm. Because no quickly effective standard treatment is available, TAE, if shown to be quickly effective, will serve as a useful treatment option in clinical practice. In that case, TAE will be practically handled as one of several standard approaches; therefore, this study will be conducted as a confirmatory study. The study will also be conducted as a single-arm confirmatory study without controls in accordance with the Research Policy of Supportive and Palliative Care in Japan [18].

Because TAE’s safety and efficacy have not been confirmed in a palliative setting, a prospective confirmatory trial is needed. Considering that palliative radiotherapy, pain killers, and TAE can be combined, we feel a randomized control study design comparing TAE with these other modalities is not necessary. In order to preserve the reliability of the results, we will evaluate efficacy and safety by maintaining strict thresholding and stopping rules.

TAE is not a major tool in this field at present. If the primary and co-primary endpoints are met, TAE can be a treatment choice for painful bone metastases, and these results will potentially provide many benefits for all eligible patients with painful bone metastases.

## Data Availability

The datasets will be available from the corresponding author on reasonable request. For data management at participating institutions, source documents and linkable anonymizing correspondence tables will be appropriately managed as the responsibility of the site investigator. Since the case report form (CRF) will be managed electronically using the electronic CRF (eCRF), the site investigator, sub-investigator, and study collaborators will pay maximum attention to security management. Study-related data will be stored at participating institutions for 5 years after end-of-study report or 3 years after all study-related publication, whichever is longer, but desirably for as long as possible. After the retention period, any study-related sample or information will be discarded only after being anonymized. After completion of the study, the raw data collection or data set created for analysis at the data center will be returned to the study representative and stored by the study representative. Auditing will be carried out by each institution’s audit system. Only clinical data managers at the central data center have access to reported case data through the EDC system during the study conduct. The data manager will transfer the final data set to the principal investigator after statistical analysis and the data will be stored in the electronic format. The findings of this trial will be submitted to an international peer-reviewed journal and the key findings will be presented at international scientific conferences.　Authorship will be ascribed in accordance with the International Committee of Medical Journal Editors guidance.

## Abbreviations

BPI-SF: Brief Pain Inventory-Short Form
CNS: Central nervous system
CRF: Case Report Form
CTCAE: Common Terminology Criteria for Adverse Events
EDC: Electronic Data Capture
JIVROSG: Japan Interventional Radiology in Oncology Study Group
J-SUPPORT: Japan Supportive Palliative and Psychosocial Oncology Group
KPS: Karnofsky Performance Status
NRS: Numeric Rating Scale
SAS: Safety Analysis Set
TACE: Transcatheter Arterial Chemoembolization
TAE: Transcatheter Arterial Embolization
